# Endothelial Response to Glucocorticoids in Inflammatory Diseases

**DOI:** 10.3389/fimmu.2016.00592

**Published:** 2016-12-14

**Authors:** Karolina A. Zielińska, Laura Van Moortel, Ghislain Opdenakker, Karolien De Bosscher, Philippe E. Van den Steen

**Affiliations:** ^1^Laboratory of Immunobiology, Rega Institute for Medical Research, KU Leuven, Leuven, Belgium; ^2^Receptor Research Laboratories, Nuclear Receptor Lab, VIB-UGent, VIB Medical Biotechnology Center, Ghent, Belgium

**Keywords:** endothelium, glucocorticoids, glucocorticoid resistance, inflammation, cytokines, tight junctions, adhesion molecules

## Abstract

The endothelium plays a crucial role in inflammation. A balanced control of inflammation requires the action of glucocorticoids (GCs), steroidal hormones with potent cell-specific anti-inflammatory properties. Besides the classic anti-inflammatory effects of GCs on leukocytes, recent studies confirm that endothelial cells also represent an important target for GCs. GCs regulate different aspects of endothelial physiology including expression of adhesion molecules, production of pro-inflammatory cytokines and chemokines, and maintenance of endothelial barrier integrity. However, the regulation of endothelial GC sensitivity remains incompletely understood. In this review, we specifically examine the endothelial response to GCs in various inflammatory diseases ranging from multiple sclerosis, stroke, sepsis, and vasculitis to atherosclerosis. Shedding more light on the cross talk between GCs and endothelium will help to improve existing therapeutic strategies and develop new therapies better tailored to the needs of patients.

## 1 Introduction

Glucocorticoids (GCs) are endogenous stress hormones with strong anti-inflammatory properties. Therefore, GCs have been therapeutically exploited for more than six decades, often at dosages, which exceed the physiological levels (1). GCs influence the function of various subtypes of immune cells including T cells, dendritic cells, macrophages, and B cells (2). A growing amount of evidence indicates that GCs also regulate multiple aspects of endothelial physiology. In this review, we discuss the effects of GCs on endothelium and the mechanisms that control GC sensitivity in endothelial cells.

## 2 Endothelial Cell Biology in a Nutshell

### 2.1 Morphology and Heterogeneity of the Endothelium

The human body contains approximately 2.5 × 10^12^ endothelial cells that are typically flat with a thin basement membrane (also known as basal lamina) enriched in type IV collagen and laminin (3–5). However, in specific blood vessels used by lymphocytes to enter the lymph nodes (high endothelial venules) endothelial cells are plum and tall with thick basal lamina (6). The thickness of endothelium varies from 0.1 µm in capillaries and veins to 1 µm in the aorta (4).

Endothelial cell phenotypes vary between different organs, between different segments of the vascular bed within the same organ, and between neighboring endothelial cells of the same organ and blood vessel type (7). In arteries and veins, the endothelium forms a continuous layer of cells held together by tight junctions (TJs)—one of the two main types of intercellular junctions in endothelium (7, 8). Endothelial cells in arteries are long and narrow and are aligned in the direction of blood flow. In contrast, in veins endothelial cells are wider and shorter and lack the alignment in the direction of blood flow (9). Capillary endothelial cells are highly adapted to underlying tissues and present many phenotypic differences between different vascular beds (9). The endothelium of capillaries may be continuous, fenestrated, or discontinuous (7). The microvascular bed of each organ is composed of specialized endothelial cells endowed with a unique set of adhesion molecules, chemokines, transcription factors, and metabolic profiles (10).

### 2.2 Functions of the Endothelium

The endothelium participates not only in blood delivery but also in organ regeneration and maintenance of homeostasis (11). Endothelial cells are metabolically active and play an important role in physiological processes such as the control of vasomotor tone, the trafficking of leukocytes between blood and underlying tissue, angiogenesis, and both innate and adaptive immunity (7).

In the central nervous system (CNS), the endothelium forms the blood–brain barrier (BBB) that controls the exchange of immune cells and mediators between blood and CNS (12). The BBB consists of a thick glycocalyx, endothelial cells linked by tight junctions (TJs), a double basement membrane, and astrocytic end-feet (13). The double basement membrane enables precise localization of sites of immune cell extravasation (14). Endothelial TJs, which constitute a crucial element of the BBB, control paracellular movement of solutes through the BBB and maintain brain homeostasis. They consist of claudins, occludin, and junction adhesion molecules (JAMs) (15). TJs are intermingled with adherens junctions (AJs) that form a belt *via* interaction between cadherins (8, 15). Proper organization of TJs requires AJs assembly (16).

### 2.3 Endothelial Activation during Inflammation and Angiogenesis

Although at rest endothelial cells present a non-reactive surface at the interface between blood and tissue, upon activation the endothelium becomes a major player in the generation of the inflammatory response (3). Pro-inflammatory cytokines produced by immune cells activate the endothelium and mediate leukocyte recruitment. Endothelial selectins such as E- and P-selectin capture leukocytes from the blood flow and mediate rolling (17). This slows down the circulating leukocytes and enables chemokines, localized on the endothelial surface, to interact with their receptors on leukocytes leading to integrin activation (12). Pro-inflammatory mediators such as tumor necrosis factor (TNF), interleukin (IL)-1, -17A, -18, -33, -36*γ*, and interferon (IFN)-*α*, -*β*, -*γ* stimulate the endothelium to produce various chemokines including CC chemokine ligand 2 (CCL2, also known as monocyte chemoattractant protein-1 or MCP-1), CCL20 (also known as macrophage inflammatory protein-3*α* or MIP-3*α*), CXC chemokine ligand 1 (CXCL1, also known as growth-related oncogene-*α* or GRO-*α*), CXCL8 (also known as IL-8), CXCL9 (also known as monokine induced by IFN-*γ* or MIG), CXCL10 (also known as IFN-*γ*-induced protein-10 or IP-10), CXCL11 (also known as interferon-inducible T cell *α* chemoattractant or I-TAC), and also integrin ligands such as intercellular cell adhesion molecule-1 (ICAM-1) and vascular cell adhesion molecule-1 (VCAM-1) (3, 18–24). In contrast, IL-10 and IL-37 decrease CXCL8 (IL-8) and ICAM-1 expression in endothelial cells (25, 26). ICAM-1 and VCAM-1 control firm adhesion and leukocyte crawling on the endothelial surface until they transmigrate through the endothelial barrier (27). Platelet endothelial cell adhesion molecule-1 (PECAM-1) controls localization of junctional proteins such as VE-cadherin and *β*-catenin and mediates leukocyte extravasation to the sites of inflammation (28).

Angiogenesis—the growth of new blood vessels—plays an important role in the control of embryonic development and is involved in various human diseases. Increased angiogenesis is associated with neoplastic diseases, while insufficient blood vessel growth limits the repair process in ischemic cardiovascular diseases (29, 30). Vascular endothelial growth factor (VEGF) regulates vascular development during embryogenesis, blood vessel formation in adults, wound healing, and organ regeneration (31, 32). In mammals, five VEGF ligands (VEGF-A, -B, -C, -D, and placental growth factor—PLGF) can bind to three receptor tyrosine kinases—VEFG receptor-1, -2, and -3 (32). Binding of VEGF to its receptor also increases vascular permeability through activation of focal adhesion tyrosine kinase (FAK) that interacts with vascular endothelial cadherin (VE-cadherin) leading to VE-cadherin–*β*-catenin dissociation (33). The Tie receptors (Tie-1 and Tie-2) and their angiopoietin ligands (ANG1–ANG4) constitute the second receptor Tyr kinase system in the vasculature. ANG–Tie controls vessel quiescence and regulates later stages of angiogenesis (30). ANG1 exerts its anti-inflammatory effects through inhibition of VEGF-induced expression of ICAM-1, VCAM-1, and E-selectin (34). Moreover, ANG1 prevents VEGF and TNF-induced expression of the pro-coagulatory molecule tissue factor (TF) (35). Later, ANG2 was described as a ligand that antagonizes ANG1 activity on Tie-2 and acts as a switch between the quiescent and inflamed state of endothelium (36, 37). ANG2 also controls later steps of leukocyte adhesion. Furthermore, ANG2 sensitizes endothelial cells toward TNF and modulates TNF-induced expression of adhesion molecules (36).

Matrix metalloproteinases (MMPs) control turnover of the extracellular matrix and are crucial in the regulation of inflammation. MMPs regulate leukocyte recruitment and modulate cytokine and chemokine activity (38, 39). For example, MMP-9 induces endothelial barrier breakdown through degradation of endothelial junctions (40). MMP-9 also plays an important role in matrix remodeling during angiogenesis and mediates influx of proteins into angiogenic tissue (41, 42).

## 3 Glucocorticoids (GCs) and the Glucocorticoid Receptor (GR)

### 3.1 GCs as Inhibitors of Inflammation

Glucocorticoids (GCs) are steroidal hormones that are synthesized in the zona fasciculata of the adrenal cortex, starting from a cholesterol scaffold (43, 44). GCs are produced in a circadian pattern or in response to stress and regulate various physiological processes including metabolism of sugar, protein, fat, muscle and bone, cardiovascular function, reproduction, and cognition (45). The hypothalamo–pituitary axis controls the secretion of cortisol, which is the physiologically active human GC (46, 47). The periventricular nucleus of the hypothalamus produces corticotrophin-releasing hormone (CRH), which induces the synthesis of adrenocorticotropic hormone (ACTH, also called corticotrophin) in the anterior pituitary gland. ACTH subsequently induces the production of the active cortisol, which in turn inhibits the secretion of CRH in the hypothalamus *via* a negative feedback mechanism (45, 48, 49).

Importantly, GCs also act as strong anti-inflammatory mediators, providing a negative feedback on inflammation (50–52). The anti-inflammatory potential of GCs has led to the application of exogenous GCs for the treatment of a variety of inflammatory disorders such as asthma, rheumatoid arthritis, and inflammatory bowel diseases (53, 54). GCs are also prescribed to transplantation patients to prevent organ rejection and used for the treatment of lymphoid cancers including leukemia, lymphoma, and myeloma (44, 51, 55).

Due to their pleiotropic actions, the use of exogenous GCs has two major drawbacks. First, high therapeutic doses used to reach desired physiological effects also induce undesired side-effects including osteoporosis, diabetes, and hypertension (48, 54, 56). Second, prolonged and/or highly dosed GC use can lead to GC resistance. This phenomenon has been discussed extensively (48, 57, 58).

### 3.2 GR Structure and Isoforms

**Figure 1:**
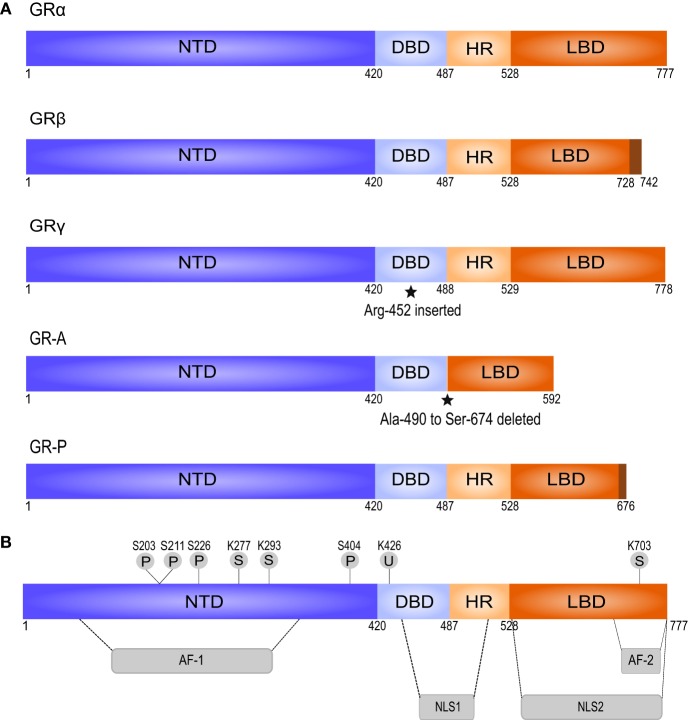
**Splice variants and posttranslational modifications of the human glucocorticoid receptor (GR)**. **(A)** The splice variant GR*β* differs from GR*α* in the C-terminal LBD, does not bind GCs, and acts as a dominant negative regulator of GR*α*. GR*γ* contains an insertion of an additional arginine residue in the DBD. This impairs its ability to regulate specific GC-responsive genes. GR-A and GR-P miss large regions in the LBD and fail to bind GCs, based on Oakley and Cidlowski (45). **(B)** GR contains several residues subjected to posttranslational modifications. AF, activation function; DBD, DNA-binding domain; HR, hinge region; LBD, ligand-binding domain; NLS, nuclear localization signal; NTD, N-terminal domain; P, phosphorylation; S, sumoylation; U, ubiquitination.

GCs exert their effects mainly by binding to the cytosolic glucocorticoid receptor (GR), a member of the nuclear receptor superfamily that is ubiquitously expressed (46, 51, 59). The human GR-coding *nr3c1* gene is located on chromosome 5. Transcription generates multiple GR isoforms due to alternative splicing (45, 48). Each isoform is composed of three major functional domains: an N-terminal domain (NTD), a central DNA-binding domain (DBD), and a C-terminal ligand-binding domain (LBD) (Figure 1). The NTD mediates the recruitment of the basal transcriptional machinery and contains the transcriptional activation function 1 (AF-1) domain with residues that undergo posttranslational modifications (PTMs) and interact with cofactors in a ligand-independent way (45, 47). The DBD contains two zinc fingers that regulate GR dimerization and DNA binding (45, 56). The DBD is linked to the LBD *via* a hinge region. The LBD forms a hydrophobic ligand-binding pocket and contains the second transcriptional activation function domain (AF-2), which is responsible for ligand-dependent interaction with coregulators and recruitment of the basal transcriptional machinery (45–47). The receptor also contains two nuclear localization signals (NLS1 and NLS2, Figure 1) at the junction of the DBD and the hinge region, and within the LBD (45).

Alternative splicing of the LBD of the human GR gene generates GR*α* and GR*β*—the best characterized isoforms of GR (45). GR*α* is the classic GR protein that mediates the majority of GCs actions. GR*β* is considered a dominant inhibitor of GR*α* that resides constitutively in the nucleus but cannot bind to GCs and has no direct effect on GC-responsive genes. However, GR*β* can antagonize the activity of GR*α* by competing for glucocorticoid response element (GRE) binding or for cofactors, or by formation of inactive GR*α*/GR*β* heterodimers (45, 48). In general, the expression of GR*β* is low but can be enhanced by pro-inflammatory cytokines (e.g., TNF and IL-1) and other immune activators (e.g., microbial superantigens) (60, 61). Increased GR*β* expression has been involved in GC resistance in a variety of inflammatory diseases (45, 50). GR*γ*, GR-A, and GR-P are the three other transcriptional GR isoforms. They are less characterized than GR*α* and GR*β*, but GR*γ* and GR-P are also known to be involved in GC resistance (62–64).

Alternative translation initiation results in further division of each isoform into subtypes. For instance, GR*α* has 8 subtypes, which are the result of 8 highly conserved start codons present in the GR mRNA. GR*α*-A is the full-length receptor and is the most abundant GR protein in many cell types, together with GR*α*-B (45). GR is also subject to various posttranslational modifications including phosphorylation, SUMOylation, and ubiquitination (43, 48, 65). Unless specified otherwise, it is always GR*α* that is referred to, since most known GC-mediated actions are exerted *via* this receptor isoform.

### 3.3 Molecular Mechanisms of GC Action

In the absence of ligand, GR mainly resides in the cytoplasm in association with chaperone proteins (e.g., hsp90, hsp70, and hsp90-binding protein p23) and immunophilins [e.g., FK506-binding protein (FKBP)-51 and FKBP-52] (45). Upon ligand binding, GR undergoes a conformational change that induces partial dissociation of the chaperone complex and exposes the nuclear localization sequences (43, 45). FKBP-51 is substituted by FKBP-52, and the transport protein dynein transports the GR complex along the cytoskeletal tracts to the nucleus (44, 65). This process also depends on importin-*α* and importin 13 (56, 57). In the nucleus, the complex dissociates and GR binds to DNA in homodimeric form (44). The cellular localization of GR is a dynamic process, since both active and inactive forms of the receptor have been demonstrated to shuttle back and forth between nucleus and cytoplasm (49, 65). Nevertheless, in most cases GR resides in the cytosol in the absence of GCs, while it translocates to the nucleus when bound to its ligand. The effect on transcription depends on binding to DNA and/or other transcription factors and on the recruitment of GR coregulators. The latter proteins include coactivators and corepressors that assist in GR transcriptional activity (66).

Binding of homodimeric GR to glucocorticoid response elements (GREs) results in transcriptional activation and rapid switches between the bound and the unbound state of GR (45, 49). This interaction induces transcription of various genes including sphingosine kinase 1 (Sphk1) and TGF-stimulated clone 22 domain protein-3 (Tsc22d3), the coding gene for GC-induced leucine zipper (GILZ) (56, 67). The latter protein is reported to bind to NF-*κ*B and AP-1, and the protective effect of this interaction has been shown in several inflammatory models (56). The promotor region of MAPK phosphatase-1 gene *mkp1* also contains a GRE site (56). The corresponding protein, MKP-1 or dual specificity phosphatase-1 (DUSP1) is a MAPK phosphatase that inactivates kinases involved in activation of AP-1—ERKs, p38, and JNK. MAPKs are also involved in the fine-tuning of NF-*κ*B signaling (56, 68). Other anti-inflammatory mediators induced by GR bound to GREs include IL-10 and annexin-1 (AnxA1) (56, 69).

Moreover, GC-bound GR can also inhibit gene transcription *via* negative GREs (nGREs) with consensus sequence CTCC(n)_0−2_GGAGA. The nature of this sequence does not allow GR dimerization, thus these inhibitory actions are mediated by monomeric GR (70). GR *via* an nGRE reduces expression of thymic stromal lymphopoietin (TSLP). The GR-dependent repression through nGRE is probably mediated *via* the assembly of a corepressor complex and the recruitment of histone deacetylases (70).

DNA-bound GR can still interact with other transcription factors *via* a mechanism called composite regulation. GR and its interacting transcription factors both bind to their own response element and influence each others’ transcription in a DNA-dependent manner (65, 68). In the hypothalamus, GR bound to an nGRE interacts with DNA-bound AP-1 and negatively regulates CRH (71).

GR can also bind to DNA-bound transcription factors, without interacting with DNA itself (65, 72). This process—called tethering—occurs at lower cortisol levels than GRE-mediated GR actions (48). However, it remains unclear if GR tethers in a monomeric or dimeric form or works in yet another fashion (73, 74). Nuclear factor kappa B (NF-*κ*B) and activator protein 1 (AP-1) belong to the best characterized proteins that are tethered by GR (44). The GR-mediated inhibition of NF-*κ*B and AP-1 represents the main mechanism for the anti-inflammatory actions of GCs, but GR can also interact with other transcription factors including interferon regulatory factor 3 (IRF3), signal transducer and activator of transcription (STAT) 3 and 5, and GATA3 (43, 65, 68, 75). GR-mediated tethering onto NF-*κ*B and AP-1 implies direct binding of presumably monomeric GR to the DNA-bound Jun- and the p65-subunit (45, 65, 68, 76); however, the surface part of GR involved in these interactions differs for AP-1 and NF-*κ*B (48). Tethering between GR and these transcription factors is reciprocal, which implies that AP-1 and NF-*κ*B can inhibit DNA-bound GR (47, 51, 68). GR can also inhibit AP-1 and NF-*κ*B through other mechanisms including the cofactor GRIP1-mediated inhibition of NF-*κ*B/IRF3 dimer formation, *via* reduced histone acetyltransferase (HAT) activity of CBP or *via* recruitment of histone deacetylase 2 (HDAC2). The latter mechanism seems to be responsible for GR-mediated inhibition of CXCL8 (IL-8) expression (55, 65, 68). GCs also repress NF-*κ*B-driven genes *via* upregulation of the cytoplasmic NF-*κ*B inhibitor (I*κ*B-*α*) (44).

Additionally, GR exerts some of its actions in a rapid non-genomic mode within minutes. Thereby GR influences signaling pathways, possibly *via* membrane-associated GR and a second messenger cascade (48, 51, 55, 77). Only recently it has been appreciated that GR elicits also pro-inflammatory pathways to prime cells for an adequate subsequent anti-inflammatory response (55).

## 4 Regulation of Endothelial Physiology by GCs

GCs exert various effects on immune cell function. GCs cause immunosuppression in pro-inflammatory T cells while increasing the activity of regulatory T cells. GCs seem to affect B cell proliferation. In macrophages, the effects of GCs are concentration-dependent and range from immunosuppression to immunostimulation. Furthermore, GCs mediate the induction of a tolerogenic phenotype in dendritic cells (2). GCs also induce apoptosis in various types of immune cells including T cells, B cells, and plasmacytoid dendritic cells (78–80). This process plays an important role in the development of the immune system and in fine-tuning of its function. Interestingly, GCs have opposite effects in macrophages and mediate their survival (81). GC-induced apoptosis also has been extensively reviewed in other cell types; however, the effects of GCs on endothelial apoptosis remain insufficiently studied (82).

**Figure 2:**
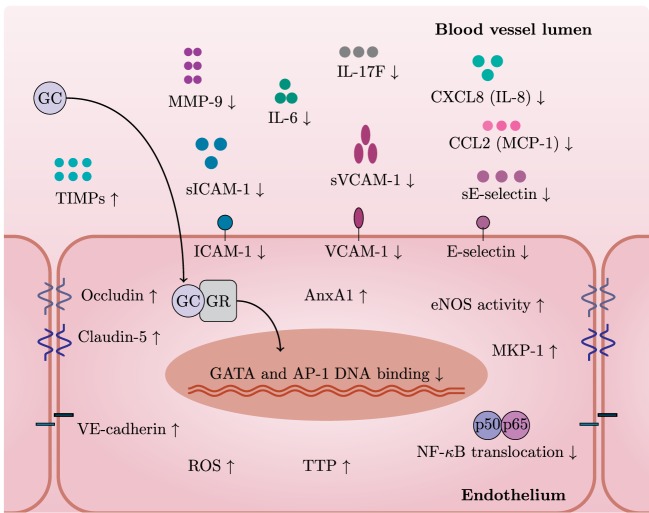
**GCs exert specific actions in endothelial cells**. In general, GCs have a variety of cell type-specific effects. This figure depicts GC actions that have been described in endothelium. After entering an endothelial cell, GCs bind to GC receptor (GR) and translocate to the nucleus. GR bound to GCs inhibits pro-inflammatory pathways by limiting GATA and AP-1 DNA binding and NF-*κ*B translocation. GCs decrease levels of adhesion molecules (VCAM-1, ICAM-1, and E-selectin) and also their soluble forms and MMP-9 while increasing levels of junctional proteins—occludin, claudin-5, and VE-cadherin. GCs induce protective molecules such as AnxA1, TTP, MKP-1, and TIMPs. Furthermore, GCs reduce levels of IL-6, IL-17F, CXCL8 (IL-8), and CCL2 (MCP-1). Stimulation with GCs increases the activity of eNOS—a critical mediator of vascular integrity. Induction of ROS represents detrimental effects of GC excess on the vasculature.

It becomes more and more clear that GCs also regulate multiple aspects of endothelial physiology (Figure 2). GCs inhibit pro-inflammatory signaling pathways in endothelium and induce protective molecules that maintain endothelial function, especially upon inflammation. For instance, dexamethasone blocks nuclear translocation of NF-*κ*B and reduces the binding of AP-1 and GATA to DNA in endothelial cells (83). GCs induce MKP-1 (also known as DUSP1), which inhibits MAPK signaling pathways, and tristetraprolin (TTP, also known as ZFP36), which destabilizes mRNAs of pro-inflammatory cytokines (84, 85). AnxA1, induced by GCs in endothelium, causes leukocyte detachment and regulates BBB integrity (86–88). Moreover, AnxA1 inhibits phospholipase A2, an enzyme that releases arachidonic acid from phospholipids to produce pro-inflammatory mediators such as prostaglandins and leukotrienes *via* cyclooxygenase (89).

GCs inhibit the endothelial production of several pro-inflammatory cytokines and chemokines including IL-6, IL-17F, CXCL8 (IL-8), and CCL2 (MCP-1) (84, 85, 90). GCs also downregulate ICAM-1, VCAM-1, and E-selectin (91–93). Moreover, GCs reduce levels of soluble forms of ICAM-1, VCAM-1, and E-selectin (sICAM-1, sVCAM-1, and sE-selectin) (94). Interestingly, GCs may also downregulate HLA-DR in IFN-*γ*-stimulated endothelial cells (92). Overall, GCs decrease leukocyte transmigration across the endothelium, thus limiting inflammation (95).

GCs also increase the activity of eNOS—a critical mediator of vascular integrity (96). Release of NO in the lumen inhibits platelet aggregation and leukocyte adhesion (97). Conversely, Iuchi et al. showed that GC excess induces reactive oxygen species and peroxynitrite formation, with possible detrimental effects on the vasculature (98).

Disruption of the endothelial barrier integrity is a common feature of various diseases including multiple sclerosis (MS) and stroke and leads to edema (99). GCs preserve endothelial barrier integrity through upregulation of junctional proteins such as occludin, claudin-5, and VE-cadherin (100–102) and down-regulation of MMP-9—an enzyme involved in junctional protein cleavage (103–105). GCs also induce endogenous MMP-9 inhibitors—TIMP-3 and TIMP-1 (103, 106). However, the induction of TIMP-1 seems controversial since contradictory results exist (106). Since MMP-9 is able to cleave CXCL8 (IL-8) and drastically potentiates its activities (107), it is tempting to speculate that downregulation of MMP-9 by GCs further affects neutrophil chemoattraction at the endothelial surface.

In summary, GCs influence endothelial barrier integrity, inhibit pro-inflammatory transcription factors, and induce protective molecules in endothelium (Figure 2). Investigation of molecular mechanisms of GC action in endothelium will enable comparison of these to the extensive data on regulation of inflammation by GC in other cell types.

## 5 Regulation of Endothelial GC Sensitivity

Although GCs have been the mainstay therapy for inflammatory diseases since 1950, GC treatment possesses significant challenges such as reduced efficacy and/or development of GC resistance (108). GC resistance has been reported in various diseases such as asthma and chronic obstructive pulmonary disease (COPD) (109) and in different cells types including peripheral blood mononuclear cells (PBMCs), B cells, and alveolar macrophages. A wide range of mechanisms causing GC resistance in these cell types have been described including increased GR*β* expression, posttranslational GR modification, impaired nuclear translocation of GR, reduced MKP-1 expression, and decreased activity of HDAC2 (58, 110–113).

**Figure 3:**
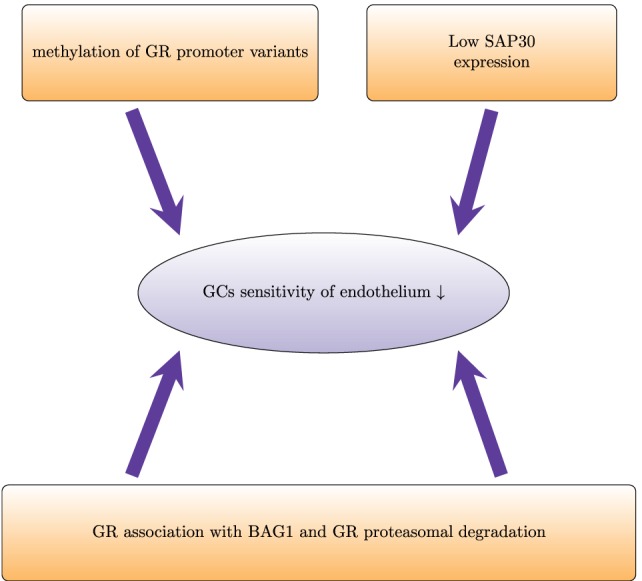
**Proteasomal degradation and epigenetic modifications regulate glucocorticoid sensitivity in endothelial cells**. Proteasomal degradation of the glucocorticoid receptor (GR) impairs GR activity and prevents physiological actions of GCs. In dexamethasone-resistant HUVECs, GR associates with the proteasomal recruiting protein BCL2-associated athanogene 1 (BAG1). This results in a shorter half-life of GR. In HUVECs, low induction of SAP30 (component of Sin3A-histone deacetylase complex) impairs GR-mediated transrepression and leads to lower GC sensitivity. Furthermore, higher methylation of the promoters of the different variants of the untranslated exon 1 of GR leads to lower expression and therefore decreased GC sensitivity.

A limited number of studies addressed GC sensitivity of endothelium uncovering several mechanisms that regulate the response of endothelium to GCs (Figure 3). In dexamethasone-resistant HUVECs, GR interacted more strongly with BCL2-associated athanogene 1 (BAG1) protein than in dexamethasone-sensitive HUVECs (114). In general, BAG1 is a cytoplasmic protein, which can translocate to the nucleus and bind to DNA, decreasing GR transactivation (115). BAG1 also interferes with GR folding through interaction with HSP70 (116). Moreover, BAG1 may target interacting proteins for proteasomal degradation (117). In HUVECs, proteasome inhibition increased GR protein levels and abolished differences between GC-sensitive and GC-resistant cells, suggesting that BAG1-mediated proteasomal degradation of GR accounts in part for the human variability in endothelial sensitivity to GCs (114). *In vivo*, proteasome inhibition improved GC sensitivity of endothelium and protected against brain edema (118).

Epigenetic mechanisms such as DNA methylation and histone modifications also regulate GC sensitivity of endothelial cells. Dexamethasone-sensitive and dexamethasone-resistant HUVECs have different GR promoter methylation patterns (119). The GR gene contains several variants of the untranslated exon 1:1A–1I (120). Each of these variants has its own promoter. Dexamethasone-sensitive cells show higher methylation levels of promoter 1D and lower methylation levels of promoter 1F. Pharmacological demethylation with 5-aza-2-deoxycytidine increased the mRNA expression of all isoforms (except 1D in resistant HUVECs) and enhanced the GC sensitivity (119). Another epigenetic mechanism that influences endothelial GC sensitivity involves Sin3A–HDAC. This multi-protein complex that regulates gene expression *via* histone deacetylation consists of SAP30, Sin3, histone deacetylases HDAC1 and HDAC2, histone-binding proteins RbAp46 and RbAp48, and other proteins. SAP30 represses gene transcription *via* tethering to gene promoters (121). Poor expression of Sap30 has been suggested as an explanation of impaired transrepression in HUVECs (122). Transgenic overexpression of SAP30 in HUVECs and analysis of its expression under inflammatory conditions would also improve the current understanding of how GC sensitivity is regulated in endothelium.

Variations in the expression of the GC-activating and -deactivating enzymes 11*β*-HSD1 and 11*β*-HSD2 have been described in endothelial cells (123). However, it remains unclear to what extent this affects the GC sensitivity.

In conclusion, the currently known mechanisms regulating GC sensitivity in endothelial cells summarized in Figure 3 include proteasomal degradation of GR and epigenetic modifications. However, the mechanisms that control GC sensitivity are complex and often cell type specific. The regulation of GC sensitivity in endothelial cells has not yet been sufficiently addressed, in particular under inflammatory conditions. Since endothelium plays a crucial role in inflammation, we believe that further studies on this subject will open up new perspectives for the development and improvement of the current treatment strategies.

## 6 Endothelial Response to GCs in Multiple Sclerosis

### 6.1 BBB Integrity in Multiple Sclerosis

Multiple sclerosis (MS) is mediated by autoreactive T cells that attack the myelin-sheath, but other inflammatory cells such as B cells, macrophages, and microglia may be as well involved in the pathogenesis. T cells infiltrate into the central nervous system (CNS) causing massive inflammation (124, 125). MS affects TJs and AJs protein expression in patients. Incubation of SVEC4–10 endothelial cells with serum from patients with MS in exacerbation phase reduced the levels of occludin and VE-cadherin (126). Claudin-5 expression was downregulated in immortalized human brain microvascular endothelial cells (TY09) after treatment with sera from the relapse phase of relapse-remitting MS (RRMS-R) or secondary progressive MS (SPMS) (127). Cytokines (TNF or IL-6) also reduced expression of TJ proteins in human brain microvascular endothelial cells (128).

### 6.2 Effects of GCs on BBB in Multiple Sclerosis

Episodes of neurological deficit (relapses) and periods of recovery (remission) characterize the most common form of MS. GCs remain standard therapy for MS (129). High-dose short-term GCs are prescribed to manage relapses although the opinions of neurologists and national guidelines on dose and duration of GC treatment vary (130, 131). In brain endothelial cells, GCs upregulate AnxA1 that regulates endothelial barrier integrity (86, 88). Indeed, AnxA1 KO mice show disrupted BBB as a result of actin cytoskeleton rearrangements (88). Junctional proteins also belong to GC target genes (132, 133). GCs induce occludin, claudin-5, and VE-cadherin in brain microvascular endothelial cells preventing endothelial barrier damage (100, 102). However, sera from patients with MS (with active disease and remission) downregulated expression of claudin-5 and occludin in cEND cells. Furthermore, dexamethasone failed to restore expression of these proteins (104). This suggests that treatment of cEND cells with sera from patients with MS impairs transactivation of junctional protein genes by GCs.

GCs downregulate expression of matrix metalloproteinase-9 (MMP-9, a key mediator of extracellular matrix remodeling and BBB disruption) in mouse brain endothelial cells incubated with sera from patients with MS (103, 104). Similarly, treatment with dexamethasone inhibits MMP-9 expression in primary rat brain endothelial cells stimulated with TNF or IL-1*β* (134). GCs also induce expression of the tissue inhibitor of metalloproteinases-1 (TIMP-1) (103).

High-dose methylprednisolone reduced expression of adhesion molecules (ICAM-1, VCAM-1) in human brain microvascular endothelial cells stimulated with TNF and in endothelial cells from patients with MS (91, 92). Methylprednisolone limited migration of PBMCs through endothelial barrier after 3 h, but this effect was less pronounced after 24 h (95). GCs also limit interactions of leukocytes with endothelium through downregulation of integrins (VLA-4, LFA-1) (135) and induction of AnxA1 that causes detachment of leukocytes from endothelial cells (87, 136).

Overall, *in vitro* studies indicate that GCs restore BBB integrity through induction of TJ proteins and protective molecules such as TIMPs. Inhibition of damaging molecules (MMP-9) and leukocyte transmigration constitute another important mode of GC action in MS.

## 7 Endothelial Response to GCs in Stroke

### 7.1 BBB Integrity in Ischemic Stroke

Ischemic stroke consists of two phases with pathological impact: ischemia and reperfusion. This induces oxidative stress in the brain and results in TJs damage and BBB disruption (15, 137). During the ischemic phase, loss of regional cerebral blood flow leads to deprivation of oxygen and nutrients in the surrounding tissue. Reperfusion reestablishes cerebral blood flow to the ischemic brain, but it also causes additional damage due to oxidative stress leading to increased blood–brain barrier permeability (15).

Oxygen and glucose deprivation and subsequent reperfusion disrupt TJs. *In vitro* experiments showed that hypoxia reduces claudin-5 levels and changes its localization in the plasma membrane in bEND.3 cells (138). MMP-2 and caveolin-1 (induced upon hypoxia) mediate occludin degradation and claudin-5 redistribution (139). Caspase-3, expressed by rat brain endothelial cells stimulated with TNF, is another enzyme involved in TJ disruption during ischemia (140, 141). Moreover, other molecules secreted in the brain such as VEGF and thrombin increase BBB permeability during stroke (142, 143).

### 7.2 Effects of GCs on BBB in Ischemic Stroke

**Figure 4:**
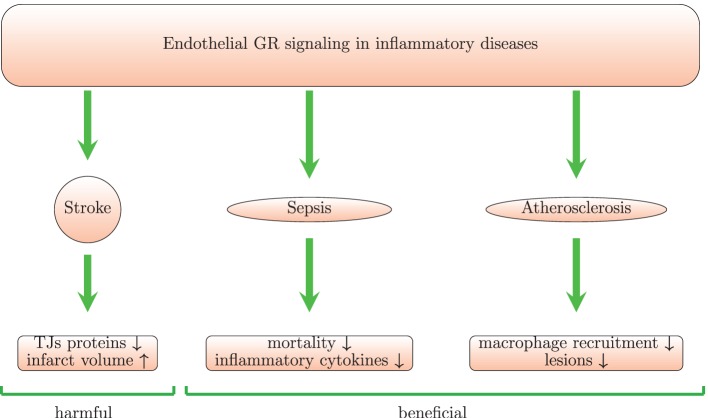
**Endothelial GR signaling is beneficial in animal models of sepsis and atherosclerosis but harmful in stroke model**. Mice with conditional GR deletion in endothelium provide a useful tool to study the role of endothelial GR signaling. In a model of stroke with these mice, it was shown that endothelial GR signaling increases infarct volume and reduces levels of junctional proteins (TJs) leading to increase in BBB permeability. In septic mice, the endothelial GR protects against LPS-induced inflammation and mortality. In an *in vivo* model of atherosclerosis, the endothelial GR reduces lesions and macrophage recruitment.

Benefits from steroid use in stroke remain controversial according to clinical trials (144, 145). *In vivo* and *in vitro* experiments provide even more contradictory data. In one study, high-dose GCs administered within 2 h of transient cerebral ischemia, increase endothelial NOS activity and reduce infarct size. The underlying mechanism for activation of endothelial NOS by GCs involves the PI3K/Akt kinases (96). However, other studies reported lack of effect (145, 146) or even damaging effects (147) of GCs in stroke. GC signaling in myeloid and endothelial cells may aggravate ischemic infarcts (Figure 4) (147). Endothelial GR signaling reduces levels of junctional proteins in stroke since Tie-2-GR KO mice (with endothelium-specific GR deletion) produce more claudin-5, occluding, and caveolin-1 than WT mice after middle cerebral brain occlusion. This suggests that endogenous GCs, released post-injury, signal through endothelial GR and reduce levels of junctional proteins. Moreover, deletion of endothelial GR reduces infarct volume (147). These data are in contrast with the classic notion that synthetic GCs induce junctional proteins [see section 4 and Ref. (132)]. Thus, the classic inductive effects of GC on TJ proteins are overshadowed and even inverted during ischemic stroke. The reasons for this discrepancy remain unclear. GR deletion abolishes the physiological effects of endogenous and exogenous GCs and could possibly also affect GC-independent processes. Therefore, the consequences of GR deletion may not be in line with described effects of high doses of exogenous GCs.

Kleinschnitz and colleagues showed occurrence of GC resistance at the hypoxic blood–brain barrier (118). Dexamethasone failed to induce junctional proteins in murine brain microvascular endothelial cells (cEND) following oxygen and glucose deprivation. Proteasome-dependent degradation of GR diminished transactivation of GC target genes. Bortezomib—a proteasome inhibitor—restored induction of junctional proteins by GCs after oxygen and glucose deprivation. Consequently, mice treated with bortezomib and GCs developed less edema (118).

In conclusion, the effects of GCs in stroke remain insufficiently understood. Beneficial, damaging effects and even GC resistance have been suggested.

## 8 Endothelial Response to GCs in Sepsis

### 8.1 Activation of Endothelium in Sepsis

Sepsis—a life-threatening organ dysfunction caused by a dysregulated host response to infection (148)—constitutes the most common cause of death in non-coronary intensive care units. The pathophysiology of sepsis remains poorly understood, and the host response mainly determines patient outcome. Alteration of endothelial cell function plays a crucial role in the pathophysiology of sepsis (149). Endothelial cells undergo excessive activation that involves release of pro-inflammatory mediators to recruit leukocytes and promote clotting to immobilize the pathogens upon tissue invasion. Functional changes observed after activation of endothelium include increased cell adhesion and leukocyte trafficking, loss of barrier function, and programed cell death (149). Cytokines such as VEGF-A, colony-stimulating factor 1 (CSF1), IL-1*β*, and TNF secreted by macrophages during tissue injury bind to receptors on endothelium resulting in endothelial activation and expression of adhesion molecules such as P-selectin, E-selectin, ICAM-1, and VCAM-1 (150, 151).

The NF-*κ*B pathway plays a central role in the induction of cytokines and adhesion molecules mentioned above (152) and mediates multiple organ inflammation in sepsis (153). Endothelial ROS-mediated NF-*κ*B and AP-1 activation as well as leukocyte recruitment in the lungs of septic mice require active MAPK kinase kinase 3 (154). Double transgenic mice that overexpress a degradation-resistant form of the NF-*κ*B inhibitor I*κ*B*α* in vascular endothelium showed endothelial selective NF-*κ*B blockade, inhibition of adhesion molecules, reduced neutrophil infiltration into different organs, and decreased endothelial permeability after LPS challenge (153). In support, another study showed that inhibition of NF-*κ*B may be beneficial to the microvasculature during endotoxemia. TNF treatment of mouse microvascular endothelial cells induced cytotoxicity and production of pro-inflammatory cytokines including IL-1*β*, IL-6, CCL5 (RANTES), and iNOS expression. NF-*κ*B inhibition abrogated these changes (155).

Endothelial cells together with leukocytes recognize pathogens *via* pattern recognition receptors that interact with microbial products (156, 157). Bacterial products such as LPS from Gram-negative bacteria interact with toll-like receptors (TLRs) leading to the activation of inflammatory and coagulation pathways (158). In endothelial cells, TLR4 may be localized intracellularly and requires then LPS internalization for its activation. The uptake system also depends on LPS binding protein and soluble CD14 (159). LPS induces CCL2 (MCP-1) secretion in activated human endothelial cells (HUVECs) through non-receptor tyrosine kinase 2 (Pyk2) activation and p38 MAPK phosphorylation (160). LPS and non-LPS components of meningococci (human pathogens that cause fatal sepsis and meningitis) can upregulate E-selectin expression in human primary endothelial cells (HUVECs) leading to neutrophil recruitment. The LPS-independent mechanism involves activation of the activating transcription factor 2 (ATF2) *via* p38 MAPK (161).

Caveolin-1 (a signaling protein associated with caveolae—small invaginations of the plasma membrane in endothelial cells) regulates the inflammatory response to LPS mediated by TLR4. After LPS exposure, caveolin-1 undergoes phosphorylation and interacts with TLR4 resulting in NF-*κ*B activation and pro-inflammatory cytokine expression in mouse lung microvascular endothelial cells. Mutation of the phosphorylation site in caveolin-1 protected mice from septic shock after LPS challenge (162). P120-catenin (p120)—a protein expressed in adherent cells—also regulates TLR4 signaling after LPS challenge. P120 blocks interaction of TLR4 with the adaptor molecule MyD88 thus inhibiting TLR4 signaling and NF-*κ*B activation in endothelial cells. In mice injected with LPS, p120 levels were inversely correlated with inflammation severity. P120 suppression in lung endothelial cells in mice with siRNA resulted in greater susceptibility to LPS and higher mortality. P120 blockade also increased ICAM-1, TNF, and IL-6 expression after LPS stimulation (163).

### 8.2 Endothelial Barrier Breakdown in Sepsis

Hyperpermeability of endothelium is a hallmark of sepsis and causes tissue edema (149, 164). ANG1 and ANG2—important biomarkers of endothelial dysfunction—bind to Tie-2 receptor on endothelial cells. Under physiological conditions, ANG1 levels are higher and trigger pro-survival and anti-inflammatory pathways in endothelium. In sepsis, ANG2 is released from Weibel–Palade bodies. ANG2 Tie-2 interaction activates pro-inflammatory, pro-thrombic pathways and vascular leakage (150, 151). Septic patients show elevated serum levels of ANG2. Incubation of human microvascular endothelial cells with serum from septic patients disrupted endothelial barrier integrity. Moreover, ANG2 alone can induce such effects and excessive systemic ANG2 levels cause pulmonary leakage in healthy mice (165). Activated protein C (APC) upregulates Tie-2 and ANG1 expression and enhances endothelial barrier function. APC treatment of HUVECs reduces permeability and upregulates the tight junction-associated protein zona occludens (ZO-1) (166).

MMPs modulate endothelial barrier integrity through processing of adherens and tight junction proteins (167). MMP-8 inhibition protects mice from death in sepsis (168–170). Arpino and colleagues showed that inhibition of metalloproteinase-mediated adherens junctions’ disruption by tissue inhibitor of metalloproteinases-3 (TIMP-3) preserves normal endothelial barrier integrity. Pulmonary microvascular endothelial cells monolayers from TIMP-3 KO mice spontaneously displayed barrier dysfunction associated with disrupted surface VE-cadherin localization (167).

Bacterial products and host inflammatory mediators are also involved in increased endothelial permeability during sepsis. The neisserial antigen fragment C2 released after proteolysis of the surface-exposed protein neisserial heparin binding antigen (NHBA) from *Neisseria meningitidis* increases endothelial barrier permeability *via* internalization of VE-cadherin. ROS induction by C2 results in VE-cadherin phosphorylation and internalization contributing to severe vascular leakage observed in meningococcal sepsis (171). TNF or LPS treatment of human dermal microvascular endothelial cells also results in endothelial barrier disruption. Soluble VE-cadherin, released by a disintegrin and metalloproteinase 10 (ADAM10) in the culture supernatant, inhibits VE-cadherin binding thus contributing to breakdown of endothelial barrier during inflammation. Inhibition of ADAM10 blocks soluble VE-cadherin production and preserves endothelial barrier integrity upon TNF or LPS stimulation. In septic patients, plasma levels of soluble VE-cadherin correlate with disease severity (172).

Stimulation of human brain microvascular endothelial cells with LPS also reduces occludin and claudin-5 levels resulting in endothelial barrier breakdown. This is mediated by LPS-induced ROS. Indeed, adenoviral expression of superoxide dismutase or inhibition of NADPH oxidase by AMP-activated protein kinase (AMPK)—an enzyme that regulates redox homeostasis in endothelial cells—abolished these effects (173). Kang and colleagues showed that heat shock protein A12B (HSPA12B) also rescues endothelial barrier integrity upon LPS stimulation in HUVECs. HSPA12B induces VE-cadherin, myosin light chain, and CDC42. Moreover, HSPA12B silencing increased lung permeability in septic mice (174).

### 8.3 GC Therapy in Sepsis

Despite over 30 years of investigation, the use of GCs in septic patients remains controversial (175, 176). Prednisolone was beneficial in a model of Gram-negative sepsis in healthy volunteers. Treatment with prednisolone decreased plasma levels of TNF, IL-6, CXCL8 (IL-8), and CCL2 (MCP-1) and increased IL-10 levels. Prednisolone also blocked neutrophil activation (177). However, short treatment with high-dose GCs was ineffective in the majority of studies performed in sepsis. Long course treatment with a low dose seems a more promising strategy (178–180). The effects of GCs on coagulation, which is also involved in sepsis, constitute another controversial issue. GCs failed to attenuate the LPS-induced coagulation cascade in a human model of sepsis (177). In contrast, Bartko and coauthors showed that GCs locally reduce coagulation in a human model of lung inflammation with LPS (181). Different experimental settings used in these studies may in part explain contradictory results. Moreover, dexamethasone has been shown to enhance the stability of TF transcript in human monocytic cells stimulated with LPS (182), whereas in HUVECs, GR knockdown increased TF expression upon LPS stimulation (183). According to a systematic review, GCs appear to upregulate the activity of coagulation factors in healthy people, whereas during active inflammation GCs decrease levels of fibrinogen and von Willebrand factor and increase plasminogen activator inhibitor-1 (PAI-1) levels (184).

Contradictory effects of GCs in sepsis may be related to the promoter polymorphism of the NF-*κ*B1 gene. Hydrocortisone failed to inhibit LPS-induced NF-*κ*B translocation in monocytes from patients with a deletion in the promoter region of NF-*κ*B1 gene (delATTG). As such, hydrocortisone treatment was also associated with increased 30-day mortality in these patients (185). This suggests that stratification of patients may help to predict the efficacy of GC treatment. This is also supported by an *in vivo* study with a sepsis model induced by cecal ligation and puncture. Stratification of mice according to the levels of circulating IL-6 not only predicted mortality but also efficacy of GC treatment, as only mice with high IL-6 responded to the GC treatment (186).

Nitric oxide (NO)-releasing derivatives of glucocorticoids show enhanced anti-inflammatory properties and may provide another pathway to improve GC therapy in sepsis (187). In particular, a NO-releasing derivative of dexamethasone, ND8008, was found to be more effective than dexamethasone in reducing the inflammatory response in LPS-stimulated mouse peritoneal macrophages and in an *in vivo* model of methicillin-resistant *Staphylococcus aureus* (MRSA) blood infection (188).

### 8.4 GR in Sepsis

Patients with septic shock exhibit high variability in GC responsiveness, and this may partially explain controversial effects of GC therapy in sepsis. Increased sickness was associated with lower GC sensitivity. However, GR*β* and 11*β*HSD1 did not influence GC sensitivity in patients with sepsis (189). GR expression and translocation gradually decrease in experimental sepsis. This may explain why early dexamethasone treatment of septic mice improved clinical outcome compared to late treatment (190). Another study showed that GCs may worsen clinical outcome in septic patients with high GR*β* levels through induction of miR-124 in T cells that downregulate GR*α* expression (191). However, other GC-regulated genes have been shown to have beneficial effects in sepsis. miR-511, induced by GCs *via* GR, confers protection *in vivo* against TNF-induced inflammation (192). In activated human CD4^+^ T cells, GCs induce miR-98 that inhibits the expression of pro-inflammatory mediators (193). Furthermore, GCs downregulate miR-155 resulting in reduction of inflammation in macrophages stimulated with LPS (194). GILZ protects SPRET/Ei mice against LPS-induced lethal inflammation (195). Pro-inflammatory cytokines and GCs, *via* GR, induce sphingosine kinase 1 (Sphk1) in a model of acute lung injury (ALI)—a complication of sepsis. This results in increase of sphingosine 1-phosphate levels (S1P), which bind to its receptor on the endothelium and triggers endothelial barrier enhancement (67). GR-mediated immunosuppression in macrophages improves survival during sepsis. Treatment of mice with a point mutation in the GR DNA-binding domain that impairs formation of transactivating GR dimers (GR*^dim^*) and mice lacking GR in macrophages with recombinant IL-1 receptor antagonist improves their survival after LPS challenge confirming that regulation of IL-1*β* in macrophages by GCs plays essential role in the control of sepsis (196). Conditional GR deletion in macrophages results in greater mortality and cytokine induction after LPS treatment (197).

### 8.5 Endothelial GR in Sepsis

Signaling through endothelial GR plays a beneficial role in models of sepsis (Figure 4). Mice with an endothelium-specific deletion of GR show increased mortality, higher levels of TNF, IL-6, and nitric oxide in comparison with control mice after challenge with LPS (183). GR deletion in HUVECs treated with LPS increases NF-*κ*B levels and IL-6 levels suggesting that endothelial GR plays a crucial role in the regulation of the NF-*κ*B pathway and nitric oxide synthesis. Moreover, mice lacking endothelial GR pretreated with dexamethasone show increased NF-*κ*B activity after LPS injection (198). A possible limitation of this study is that GR deletion is driven by the Tie-1 promoter, which may be not fully endothelium-specific, as hematopoietic stem cells also express Tie-1 (199). Although GR-mediated induction of I*κ*B*α* plays the main role in GC-induced suppression of NF-*κ*B in monocytes and lymphocytes, in endothelial cells physical interaction between GR and NF-*κ*B seems more important (200). Brostjan and colleagues also found that dexamethasone inhibits NF-*κ*B-mediated transcription of E-selectin in porcine endothelial cells stimulated with LPS or TNF (93). Prednisolone reduced levels of soluble E-selectin and VCAM-1, respectively, in a human endotoxemia model (94). Maximal inhibition was achieved at 30 mg. However, levels of soluble ICAM-1 were not affected by the treatment (94). Interestingly, GCs also decreased LPS-induced expression of IL-17F *in vitro* in rat pulmonary endothelial cells and *in vivo* in a model of LPS-induced ALI. This was paralleled by inhibition of the lung injury (90).

In summary, endothelial GR signaling protects against LPS-induced sepsis (Figure 4). GCs also inhibit endothelial activation in *in vitro* and *in vivo* models of sepsis.

## 9 Endothelial Response to GCs in Vasculitis

### 9.1 Activation of Endothelium during Vasculitis

Vasculitis is a heterogeneous group of disorders characterized by self-sustaining inflammation of blood vessels. Small vessel vasculitis affects venules and capillaries and is a hallmark of immune-complex vasculitis or necrotizing vasculitis. Giant cell arteritis and Takayasu’s arteritis involve medium and large vessels (201). Diverse triggers such as bacterial and viral infections can cause vasculitis, but it can also occur as a primary disease of unknown origin (202, 203).

Activation of endothelium and leukocyte infiltration are hallmarks of vasculitis and are tightly interrelated. Anti-endothelial antibodies (AECA) constitute an important cause of endothelial activation. Stimulation of human kidney endothelial cells with AECA upregulated vascular adhesion protein-1 (VAP-1), CCL2 (MCP-1), MHC class I-related antigen A (MICA), and CXCL6 (GCP-2) expression. AECA also activated c-Jun, ATF2, and NF-*κ*B (204). IgA anti-endothelial cell antibodies present in sera from children with active Henoch–Schönlein purpura induce ERK phosphorylation and CXCL8 (IL-8) expression in HUVECs (205).

Furthermore, several cytokines and enzymes in the plasma of vasculitis patients contribute to endothelial activation. Plasma from patients with Kawasaki disease [acute febrile childhood vasculitis characterized by the development of coronary artery abnormalities in 25–30% of untreated patients (206)] activates HUVECs and induces MMP-9 expression. This effect is counteracted by IFN-*γ* (207). Another study showed that sera from patients with acute Kawasaki disease induce ICAM-1 expression in HUVECs (208).

TNF-like weak inducer of apoptosis (TWEAK) serum levels of patients with acute Henoch–Schönlein purpura (the most common systemic vasculitis in children) are increased and correlate with severity of the disease. TWEAK induces CCL5 (RANTES) and CXCL8 (IL-8) expression in a human dermal endothelial cell line (209). TWEAK also upregulates E-selectin and ICAM-1 as shown *in vitro* and in an *in vivo* model of cutaneous vasculits (210).

Proteinase 3 (PR3), which is mainly produced by neutrophils and is the main autoantigen in granulomatosis with polyangiitis, binds also to endothelium and induces CXCL8 (IL-8) and CCL2 (MCP-1) that provide chemotactic signals for neutrophils and monocytes. PR3 also upregulates the expression of adhesion molecules such as ICAM-1 on endothelial cells (211, 212).

Among the signaling pathways induced in endothelial cells during vasculitis, NF-*κ*B and endoplasmic reticulum stress response protein X-box binding protein-1 (XBP-1) appear important. In a local Shwartzman reaction model of TNF-induced vasculitis, XBP-1 contributes to the vascular damage as shown in mice with an endothelium-specific XBP-1 deletion (213). This transcription factor can be induced by TNF and upregulates the p65-subunit of NF-*κ*B, resulting in sustained NF-*κ*B-mediated transcription of pro-inflammatory molecules. XBP-1 also regulates leukocyte adhesion to endothelium *in vitro* and neutrophil infiltration *in vivo* (213).

### 9.2 Endothelial Injury in Vasculitis

Endothelial injury in vasculitis involves detachment of whole endothelial cells and apoptosis. These processes play an important role in the pathogenesis of vasculitis. Regulation of the balance between endothelial injury and endothelial repair is poorly studied. In children with active vasculitis the levels of VEGF, ANG2, circulating endothelial cells (CECs), and endothelial progenitor cells (EPCs) increase (214). EPCs migrate from bone marrow to the lesions. Chronic inflammation during vasculitis may impair EPC function and reduce endothelial repair capacity (215).

In a mouse model of Kawasaki disease, activation of Nlrp3 inflammasome results in endothelial injury (216). In small vessel vasculitis, neutrophil-derived myeloperoxidase contributes to endothelial injury. It causes loss of cell membrane integrity and morphological changes in endothelial cells (217). The transfer of myeloperoxidase from neutrophils to endothelial cells is cell contact dependent and is mediated by *β*2 integrin (218).

Bacterial products constitute another group of agents involved in endothelial injury during vasculitis. *Haemophilus somnus* infection often causes vasculitis and thrombosis in bovines (219). This pathogen and its lipooligosaccharide induce apoptosis in bovine pulmonary artery endothelial cells *in vitro* (220).

### 9.3 GC Therapy in Vasculitis

GC therapy remains the golden standard in vasculitis (221, 222). GCs are used to induce remission and as maintenance therapy (223). Antineutrophil cytoplasmic antibody (ANCA)-associated vasculitis (AAV) is a group of unique diseases characterized by necrotizing inflammation of small blood vessels and presence of ANCA (224). GCs constitute the main therapy for AAV due to their rapid onset of action (225) and are an effective treatment for non-severe relapses in the majority of patients with AAV (226). However, there is no consensus among clinicians on induction doses and therapeutic schedules of GC therapy in AAV to induce and maintain remission (225). Patients with systemic necrotizing vasculitis who do not respond to a sole GC treatment are prescribed GC combined with cytotoxic agents (227).

In patients with giant cell arteritis, treatment with high-dose GCs reduced neutrophil adhesion to endothelium already after 48 h. However, 6 months after the GC dose the suppressor phenotype of neutrophils was less pronounced (228). Another study showed that GCs inhibit IL-17 release, but IFN-*γ* levels remained unaffected (222). A plethora of cytokines was identified in vasculitic lesions in giant cell arteritis with two dominant clusters: IL-6/IL-17 and IL-12/IFN-*α*. The IL-6/IL-17 cluster, active in early and untreated disease, is highly sensitive to GCs. Despite this inhibition, inflammation persists in patients with elevated levels of IL-12 and IFN-*γ* that are resistant to GCs (229). High doses of GCs inhibited IL-2 and IFN-*γ* from T cells, but IFN-*γ* mRNA levels were only slightly affected in severe combined immunodeficiency (SCID) mice engrafted with human temporal arteries. Moreover, activation of macrophages was only partially inhibited by GCs in this model, suggesting a strong GC-resistant component (230).

### 9.4 Suppression of Endothelial Response by GCs in Vasculitis

Endothelial activation clearly plays a prominent role in the vascular damage and overall pathophysiology of the various vasculitides. Although GCs have in general multiple beneficial effects on endothelial cells (see section 4), it remains unclear to what extent the therapeutic effects of GCs in vasculitis are mediated through the endothelium. For instance, one study indicated that GCs improve flow-mediated dilation, a marker of endothelial function in giant cell arteritis patients (231), but a later and larger study failed to obtain statistical significance of this parameter (232). In an *in vitro* model of vasculitis with HUVECs cocultured with neutrophils and stimulated with TNF or IL-1*β*, GCs inhibited E-selectin expression, which is involved in the damaging hyperadhesiveness of neutrophils to endothelium (233). Another study showed that dexamethasone inhibits IL-6 production and E-selectin expression in human coronary arterial endothelial cells stimulated with TNF (234).

Overall, more *in vivo* studies are needed to delineate the endothelial response and sensitivity to GCs in vasculitis. For instance, experiments with mice with endothelium-specific deletion of the GR may yield interesting insights.

## 10 Endothelial Response to GCs in Atherosclerosis

### 10.1 Activation of Endothelium during Atherosclerosis

Atherosclerosis affects large vessels and is the primary cause of heart disease and stroke. High-fat, high-cholesterol diet causes accumulation of lipoprotein particles and their aggregates in the intima at the lesion sites of predilection (235). Low density lipoproteins (LDL) passively diffuse through TJs and accumulate in the subendothelial matrix. Oxidized LDL stimulate the endothelium to produce adhesion molecules and reduce NO production, which is a critical mediator of vasorelaxation (235).

In animal models, endothelial cells in the arteries express VCAM-1 in response to cholesterol accumulation in the intima resulting in monocyte recruitment (236). Monocytes subsequently transmigrate into the intima, where they take up lipoproteins forming foam cells (235). Smooth muscle cells also express VCAM-1 that promotes recruitment and retention of mononuclear cells in the intima (237). Several pro-inflammatory mediators produced by endothelial cells play an important role in recruitment of immune cells (236). CCL2 (MCP-1) promotes monocyte recruitment and is the dominant mediator of macrophage accumulation in atherosclerotic lesions (238). CCR2 KO mice developed less atherosclerotic lesions (239). Macrophage migration inhibitory factor (MIF)—a key regulator in chronic and acute inflammation—also plays a role in the pathogenesis of atherosclerosis and is upregulated in monocytes and endothelial cells in human atherosclerotic lesions (240, 241). MIF blockade results in plaque regression and lower monocyte and T cell content in the lesions (242). Tissue factor (TF) triggers coagulation and its elevated levels are found in atherosclerotic plaques. IL-33 induces TF expression in HUVECs and coronary artery endothelial cells in a ST2 and NF-*κ*B-dependent manner. In human carotid atherosclerotic plaques, TF levels positively correlate with IL-33 expression (243). Vascular endothelial cells and macrophages produce also T cell attractants CXCL9 (Mig), CXCL10 (IP-10), and CXCL11 (I-TAC). These chemokines play an important role in the recruitment of T cells within the lesions, since activated T cells express the corresponding receptor CXCR3 (244).

Bacterial infection-mediated inflammation has been shown to facilitate development of atherosclerosis *via* the following mechanism: flagellin (principal component of bacterial flagellum) induces interaction between NADPH oxidase-4 (Nox4) and TLR5 (245). Nox4 seems unusual because it releases H_2_O_2_ in contrast to other Nox family members that produce superoxide (246). Nox4 and TLR5 interaction results in H_2_O_2_ generation and induction of CXCL8 (IL-8) and ICAM-1 in human aortic endothelial cells. Nox4 deficiency results in resistance to flagellin-induced atherosclerosis in ApoE KO mice (245). *Porphyromonas gingivalis*, a bacterial species that causes periodontitis, impairs tube formation and induces adhesion molecules (ICAM-1, VCAM-1) in human coronary artery endothelial cells through TLR4 signaling (247).

### 10.2 The Role of Endogenous GCs in Atherosclerosis

The link between GCs and the cardiovascular system is complex. Epidemiological models showed correlation between endogenous plasma corticosteroid levels and severity of cardiovascular disease (248). The function of endogenous GCs in the development of atherosclerosis remains unclear. Adrenalectomy stimulates atherosclerosis in LDL receptor KO mice (249), but not in ApoE KO mice (250). Fine-tuning of GCs signaling by 11*β*-HSD1 (converts inactive GCs into active form) and 11*β*-HSD2 (converts active GCs into inactive form) also plays a role in atherosclerosis. Inhibition of 11*β*-HSD1 in atherosclerosis-prone apoE KO mice directly attenuates atherosclerotic plaques and decreases pro-inflammatory gene expression in the vasculature (251). 11*β*-HSD1 KO mice on apoE KO background challenged with western diet showed smaller plaques and lower macrophage content. Foam cell formation was decreased as well as expression of several TLRs (252). 11*β*-HSD1 inhibition reduces T cell infiltration, CCL2 (MCP-1) and VCAM-1 levels in atherosclerotic plaques (253).

### 10.3 Synthetic GCs in Atherosclerosis

The therapeutic effects of GCs in atherosclerosis development remain unclear. Mice with human-like lipoprotein metabolism treated with corticosterone showed a decrease in the total atherosclerotic lesion area and in macrophage content in the plaques. However, the treatment negatively affected body fat metabolism: it increased body weight, subcutaneous adipose tissue, and food intake (254). Therefore, a localized treatment would be beneficial, since this could help to avoid the systemic side-effects. This remains challenging because of the difficulty in implanting drug-eluting devices without subjecting vessels to mechanical damage. Kastrup and colleagues developed a drug-eluting bioadhesive gel that can be glued onto the inside surface of blood vessels (255). Atherosclerotic mice treated with this steroid-eluting adhesive gel show lower macrophage content, less plasma cytokines, and biomarkers of inflammation in the plaques.

Importantly, atherosclerosis may even occur as a side effect of GC therapy. For instance, in patients with rheumatoid arthritis GC therapy was associated with carotid plaque, arterial incompressibility, and dose-dependently also with all-cause and cardiovascular mortality (256, 257). As a possible explanation, GCs induce macrophage adipocyte fatty acid-binding protein (FABP4), a pro-atherogenic protein upregulated by long chain fatty acids and oxidized LDL. Pitavastatin is a HMG-CoA reductase inhibitor that lowers cholesterol levels and therefore also FABP4 levels. When combined with dexamethasone pitavastatin enhances in a synergistic manner FABP4 expression (258). Furthermore, co-treatment of apoE KO mice (challenged with high-fat diet) with dexamethasone and pitavastatin exacerbates diet-induced atherosclerotic lesions (259).

Although synthetic GCs reduce inflammation in animal models of atherosclerosis, metabolic side-effects may occur and overshadow beneficial effects. Localized GC treatment may therefore prove more effective and deserve further investigation.

### 10.4 Effects of GCs on Endothelium in Atherosclerosis

Endothelial GR suppresses atherogenesis in animals and plays an important role in the atheroprotective actions of endogenous GCs (Figure 4). ApoE KO mice lacking endothelial GR subjected to a high-fat diet developed more severe atherosclerotic lesions and showed increased macrophage recruitment (260).

GCs exert various anti-inflammatory actions such as inhibition of VCAM-1 and CCL2 (MCP-1) (see section 4) *via* endothelial GR. Two GC-inducible genes tristetraprolin (TTP, also known as ZFP36) and glucocorticoid-induced leucine zipper (GILZ, also known as TSC22 domain family protein-3) influence endothelial cell function during atherosclerosis (261, 262). GILZ is a key endogenous regulator of the immune response that interacts with signal transduction pathways (1). Degenerated aortocoronary saphenous vein bypass grafts (with inflammatory cell activation) showed lower protein and mRNA levels of GILZ in comparison with healthy controls. GILZ was also downregulated in HUVECs and macrophages upon TNF stimulation, and GILZ KO enhanced pro-inflammatory gene expression (261). TTP destabilizes pro-inflammatory cytokine mRNA through binding to AU-rich elements within their 3′-untranslated regions (263). LPS, GCs, and forskolin induce TTP expression in human aortic endothelial cells. Endothelial cells overlying atherosclerotic lesions in mice and humans express TTP that has been shown to reduce NF-*κ*B activation during atherosclerosis (262).

In conclusion, the main protective effects of GCs on endothelium during inflammation include inhibition of pro-inflammatory molecules (e.g., VCAM-1 and MCP-1) and induction of protective molecules (such as GILZ and TTP). Moreover, endothelial GR protects against atherosclerosis in *in vivo* models. However, the overall effects of GCs in atherosclerosis are complex and also include deleterious metabolic effects.

## 11 Conclusion

A plethora of *in vivo* and *in vitro* studies have shown that the endothelium represents an important target for GCs. GCs induce junctional proteins and several protective molecules in endothelium. Furthermore, GCs inhibit endothelial expression of pro-inflammatory mediators such as cytokines, chemokines, and adhesion molecules. This greatly contributes to the beneficial effects of GCs in various inflammatory diseases, because the endothelium is a crucial player in inflammation. However, the beneficial actions of GCs may sometimes be overshadowed by detrimental side-effects and GC resistance, in particular on long-term treatment. GC resistance often arises as a consequence of disease in otherwise GC-sensitive individuals and tissues and is a major problem in clinical practice. Although various mechanisms, including proteasomal degradation and epigenetic modifications, have been reported to modulate GC sensitivity of endothelium, it appears that the current knowledge about the regulation of GC sensitivity in endothelial cells is still lagging behind our understanding of GC-mediated effects in other cell types such as macrophages and dendritic cells.

As future perspectives, we believe that further studies should be performed to investigate the precise interactions between GC signaling and pro- and anti-inflammatory pathways in endothelial cells. Such interactions are often cell type-specific and differ according to the cytokines and other stimuli used to activate the endothelial cells *in vitro* or according to the disease *in vivo*. Furthermore, exploring the regulation of GR activity through the variety of its isoforms and posttranslational modifications and by non-coding RNAs in endothelial cells is highly promising. These mechanisms fine-tune the interactions of GR with other signaling molecules, transcription factors, and cofactors and may dictate GC sensitivity and resistance. Finally, the variability of endothelial GC-mediated signaling between individuals and between endothelial cells within the body has to be addressed, since such information will help to define which diseases and which patient groups will benefit the most from GC therapy. Overall, this is a formidable task, but it will yield great benefits for fundamental science, pharmaceutical development, and precision medicine for patients.

## Author Contributions

KZ: design and writing of all sections and figures except section 3 on GCs and GR. LVM: writing of section 3 on GCs and GR. KDB: design and writing of section 3 on GCs and GR and critical reading. GO: conception and design of the review and critical revision of text and figures. PVdS: conception, design, and participation in the writing of the review and critical revision of text and figures.

## Conflict of Interest Statement

The authors declare that the research was conducted in the absence of any commercial or financial relationships that could be construed as a potential conflict of interest.
